# Profibrotic role of the SOX9–MMP10–ECM biosynthesis axis in the tracheal fibrosis after injury and repair

**DOI:** 10.1016/j.gendis.2023.06.012

**Published:** 2023-07-15

**Authors:** Lei Gu, Anmao Li, Chunyan He, Rui Xiao, Jiaxin Liao, Li Xu, Junhao Mu, Xiaohui Wang, Mingjin Yang, Jinyue Jiang, Yang Bai, Xingxing Jin, Meiling Xiao, Xia Zhang, Tairong Tan, Yang Xiao, Jing Lin, Yishi Li, Shuliang Guo

**Affiliations:** a Department of Respiratory and Critical Care Medicine, The First Affiliated Hospital of Soochow University, Suzhou 215006, China; b Department of Respiratory and Critical Care Medicine, The First Affiliated Hospital of Chongqing Medical University, Chongqing 400016, China; c Department of Infection Disease, The First Affiliated Hospital of Wenzhou Medical University, Wenzhou, Zhejiang 325000, China

**Keywords:** Extracellular matrix deposition, Fibroblast activation, MMP10, SOX9, Tracheal fibrosis

## Abstract

Fibroblast activation and extracellular matrix (ECM) deposition play an important role in the tracheal abnormal repair process and fibrosis. As a transcription factor, SOX9 is involved in fibroblast activation and ECM deposition. However, the mechanism of how SOX9 regulates fibrosis after tracheal injury remains unclear. We investigated the role of SOX9 in TGF-β1-induced fibroblast activation and ECM deposition in rat tracheal fibroblast (RTF) cells. SOX9 overexpression adenovirus (Ad-SOX9) and siRNA were transfected into RTF cells. We found that SOX9 expression was up-regulated in RTF cells treated with TGF-β1. SOX9 overexpression activated fibroblasts and promoted ECM deposition. Silencing SOX9 inhibited cell proliferation, migration, and ECM deposition, induced G2 arrest, and increased apoptosis in RTF cells. RNA-seq and chromatin immunoprecipitation sequencing (ChIP-seq) assays identified MMP10, a matrix metalloproteinase involved in ECM deposition, as a direct target of SOX9, which promotes ECM degradation by increasing MMP10 expression through the Wnt/β-catenin signaling pathway. Furthermore, *in vivo*, SOX9 knockdown ameliorated granulation proliferation and tracheal fibrosis, as manifested by reduced tracheal stenosis. In conclusion, our findings indicate that SOX9 can drive fibroblast activation, cell proliferation, and apoptosis resistance in tracheal fibrosis via the Wnt/β-catenin signaling pathway. The SOX9–MMP10–ECM biosynthesis axis plays an important role in tracheal injury and repair. Targeting SOX9 and its downstream target MMP10 may represent a promising therapeutic approach for tracheal fibrosis.

## Introduction

Tracheal fibrosis, caused by tracheal mucosal scarring or submucosal tissue hyperplasia, is a pathological process that can cause the airway to become smaller and result in breathing difficulty.^1^ The main clinical manifestation of tracheal fibrosis is dyspnea, and the degree of dyspnea is related to the degree of stenosis caused by granulation tissue or scar tissue hyperplasia. Normally, α-SMA positive cells are activated fibroblasts or myofibroblasts that are essential for tissue repair, and their excessive activation is causatively associated with fibrosis. Abnormally activated fibroblasts, derived from interstitial fibroblasts, epithelial–mesenchymal transformation (EMT), or circulating fibroblasts, play a leading role. EMT plays an initiating role in fibrotic diseases, where excessive deposition of the extracellular matrix (ECM) leads to fibrosis, followed by tissue hardening and/or scarring.^2^ Activated fibroblasts are highly proliferative, can secrete excessive ECM—which is invasive and migratory—and are resistant to apoptosis.^3^ The excessive replacement of ECM in the parenchyma will eventually lead to organ dysfunction due to the formation of permanent fibrous scar tissue, and can even lead to patient death.^4^

Currently, there are few studies on the molecular mechanisms underlying tracheal fibrosis. As a transcription factor, SOX9 regulates multiple cellular processes, including fibrosis and tumor growth. It has been found that SOX9 plays an important role in fibrosis of the liver, heart, kidneys, and lungs.^5^, ^6^, ^7^, ^8^, ^9^ In a study of pulmonary disease-related fibrosis, repeated injury and repair of alveolar epithelial cells was the main cause of functional pulmonary parenchyma destruction, and nonfunctional connective tissue (fibrosis) gradually replaced normal tissue, which led to the loss of alveolar function, eventually leading to respiratory insufficiency and early death.^10^^,^^11^ Analysis of lung tissues of patients with idiopathic pulmonary fibrosis (IPF) and human lung fibroblasts revealed that the most significant regulatory genes related to metabolism, growth, and cell division in fibroblasts were mainly located in the ECM.^12^ A recent study from our lab has shown that SOX9 is up-regulated in the formation of tracheal granulation tissues in rat models, and SOX9 knockdown can alleviate tracheal fibrosis following brushing by preventing fibroblast inactivation and ECM deposition.^13^ Therefore, we believe that SOX9 is involved in recurrent granulation tissue proliferation after tracheal injury, and the inhibition of SOX9 may be the key to solving this problem. However, the mechanism through which SOX9 regulates tracheal fibrosis remains unclear.

Studies have shown that SOX9 can promote tracheal fibrosis; however, there are no existing experiments to study how SOX9 affects fibroblast activation and regulates tracheal repair and fibrosis after injury. Therefore, we aimed to study the specific mechanism of SOX9 in tracheal fibroblasts and ECM deposition to provide a theoretical basis for the prevention and treatment of tracheal fibrosis through *in vitro* and *in vivo* experiments.

## Materials and methods

The descriptions of RNA isolation and reverse transcription-quantitative polymerase chain reaction (RT-qPCR), Western blotting (WB), immunofluorescence staining, flow cytometry, wound-healing assay, and 5-ethynyl-2′-deoxyuridine (EdU) staining were presented in the Supplementary Methods.

### Cell culture, transfection, and treatment

Primary rat tracheal fibroblasts (RTFs) were derived as described previously.^14^^,^^15^ Vimentin (ab92547; Abcam, Cambridge, UK)-positive cells were considered as RTFs. Cells were cultured with DMEM (Gibco, Waltham, MA, United States) supplemented with 20% fetal bovine serum (Gibco) at 37 °C in 5% CO_2_. RTF cells from passages 2 to 5 were used in subsequent experiments. Small interfering RNAs targeting SOX9 (si-SOX9) or negative control (si-NC) were obtained from Tsingke Biotechnology Co., Ltd., Beijing, China. The overexpression adenovirus targeting SOX9 (Ad-SOX9) and negative control (Ad-NC) was a gift from Professor Huang Wei of Chongqing Medical University (Chongqing, China). Cells (5 × 10^4^) were cultured in a 6-well plate for 24 h and then transfected with si-NC or si-SOX9. The efficiency of SOX9 knockdown was verified by RT-qPCR and WB. SOX9 overexpression was verified by WB 48 h post-transfection. RTFs were divided into different groups as follows: (i) control group and TGF-β1 (0, 3, 5, 10 ng/mL for 48 h and 10 ng/mL for 0, 24, and 48 h) group. TGF-β1 (10 ng/mL for 48 h) was used for follow-up experiments. (ii) si-NC, si-NC + TGF-β1, si-SOX9, and si-SOX9 + TGF-β1 groups. (iii) Ad-NC + TGF-β1 and Ad-SOX9 + TGF-β1 groups. Recombinant human TGF-β1 was purchased from Peprotech (Rocky Hill, NJ, United States).

### Chromatin immunoprecipitation sequencing (ChIP-Seq)

The ChIP assay was performed on RTF cells. The 10% lysis-sonicated chromatin was stored and named “input”, 80% was used in immunoprecipitation reactions with anti-SOX9 antibody (ab185230; Abcam) and named “IP”, and 10% was incubated with rabbit IgG (Cell Signaling Technology, Danvers, MA, USA) as a negative control and named “IgG”, respectively. ChIP-seq libraries were constructed, and fragments corresponding to 200–500 bp were enriched, quantified, and sequenced on a DNBSEQ-T7 sequencer (MGI Tech Co., Ltd., Shenzhen, China). Gene ontology (GO) analysis and Kyoto Encyclopedia of Genes and Genomes (KEGG) enrichment analysis for annotated genes were implemented using KOBAS software (version.3.0.3; Beijing, China).

### ChIP-quantitative PCR assays (ChIP-qPCR)

ChIP-qPCR was performed as previously described.^16^ Anti-Sox9 rabbit antibody (ab185230; Abcam) was used for the immunoprecipitation of Sox9-bound chromatins. The primer pair used to detect the SOX9 binding site for the MMP10 ChIP 1 sequence was F 5′AAACAATCCTGGGAGGCACA3′, R 5′TCCTTTATTGGTGATGGCACAT3′; that for the ChIP 2 sequence was F 5′TACTAGCCCTTCAGGTTAGGAGTC3′, R 5′GGATCTCAGGGGCAGAAGGTAC3′; and that for the ChIP 3 sequence was F 5′ACCTTCTGCCCCTGAGATCC3′, R 5′AACCAAACGGGTGAAGGAAT3′.

### Plasmid construction and dual-luciferase reporter assay (dual-LUC assay)

SOX9 was amplified from 293T cells using PCR and cloned into the pcDNA™3.1(+) vector. The MMP10 promoter-luciferase reporter plasmids were constructed using the pGL3 vector. Hieff Trans™ Liposomal Transfection Reagent was used for gene delivery, and a dual-LUC reporter assay was performed according to the manufacturer's instructions (YEASEN, Shanghai, China).

### Tissue histopathology (immunohistochemistry staining/IHC) of tracheal fibrosis in rat models

The modeling process was improved based on previous research.^13^ All rats were purchased from Chongqing Medical University, and all animal use programs were approved by the Animal Care and Use Committee of the First Affiliated Hospital of Chongqing Medical University (No. 2022-112). Five rat tracheal tissue samples from the LV-shRNA-NC + model group and five from the LV-shRNA-SOX9 + model group were collected for RNA-seq and IHC analysis. Differentially expressed genes identified by RNA-seq and CHIP-seq were selected for Venn diagram analysis and protein–protein interaction (PPI) network diagram construction. IHC staining was performed according to the manufacturer's instructions (cat. SP-9001; ZSGB-BIO, Beijing, China). After antigen microwave thermal retrieval, endogenous peroxidase blocking, and blocking with normal goat serum, the samples were incubated with the primary antibody against SOX9 (cat. ab185966; 1:200; Abcam), MMP10 (cat. DF7152; 1:100; Affinity), or TIMP1 (cat. WL02342; 1:100; Wanleibio, Shenyang, China) at 4 °C overnight, and then with the corresponding secondary antibody for 30 min. The sections were developed using diaminobenzidine, counterstained with hematoxylin, and mounted.

### Statistical analysis

GraphPad Prism V7.0 (GraphPad Software, San Diego, CA) was used for the statistical analyses. The values were expressed as the means ± standard deviation (SD). The tests for normal distribution and homogeneity of variance were conducted. The two groups were compared using an independent-sample *t*-test. Multiple groups were compared via analysis of variance. A *P*-value <0.05 was considered statistically significant.

## Results

### SOX9 expression and ECM deposition are increased in TGF-β1-induced RTFs

**Figure 1:**
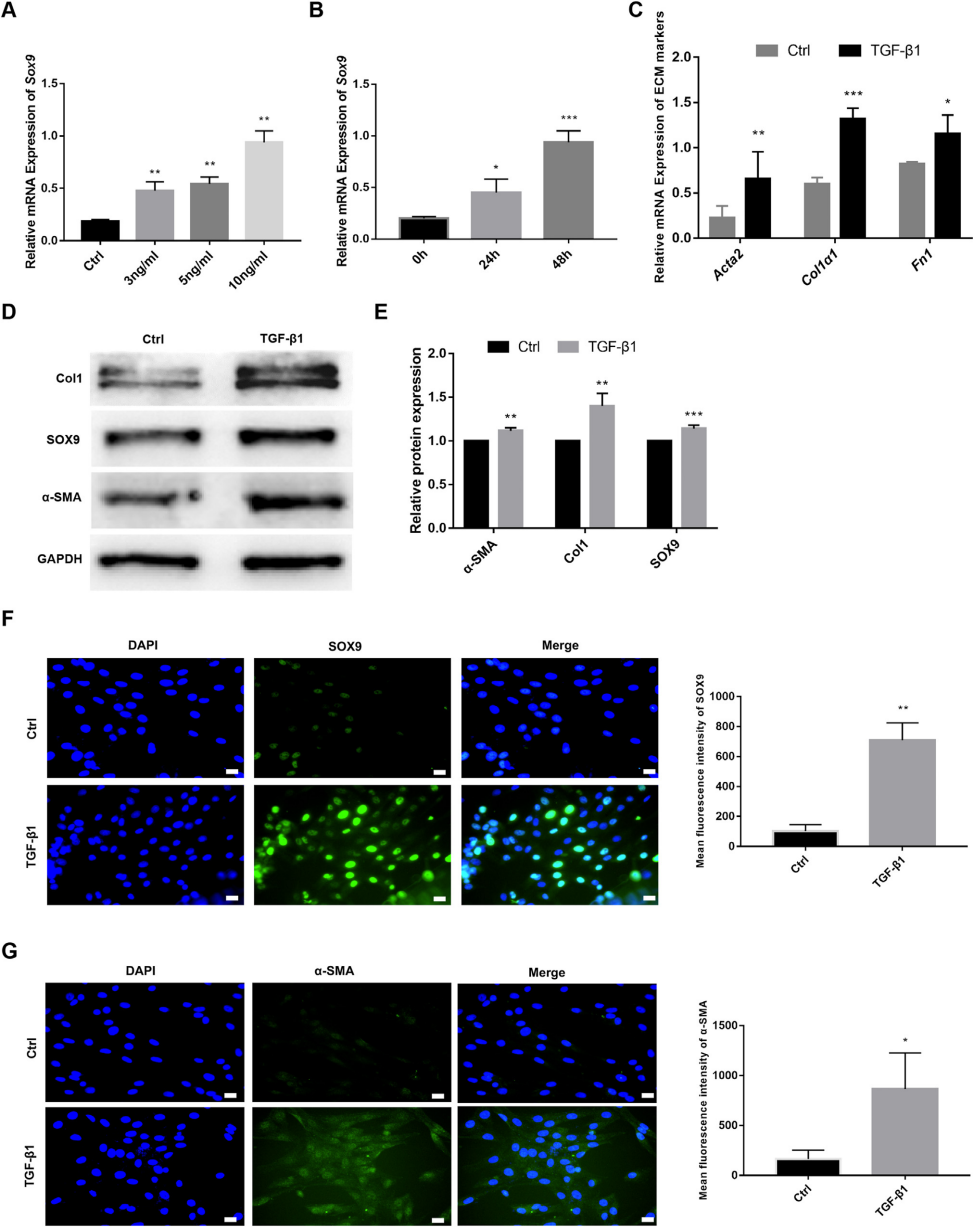
SOX9 expression and ECM deposition are increased in TGF-β1-induced rat tracheal fibroblasts (RTFs). **(A)** RTFs were treated with different concentrations (0, 3, 5, and 10 ng/mL) of TGF-β1 for 48 h. The relative mRNA expression of SOX9 was measured by RT-qPCR. **(B)** RTFs were treated with 10 ng/mL TGF-β1 for 0, 24, and 48 h. The relative mRNA expression of SOX9 was measured by RT-qPCR. **(C)** The relative mRNA expression of ECM markers (*Acta2*, *Col1α1*, and *Fn1*) measured by RT-qPCR. **(D)** The protein expression of collagen 1, SOX9, and α-SMA measured by Western blot. **(E)** Quantitative analysis of (D). **(F,G)** Immunofluorescence staining of SOX9 and α-SMA. Scale bars: 20 μm. RTF cells were treated with 10 ng/mL TGF-β1 for 48 h. ^∗^*P* < 0.05, ^∗∗^*P* < 0.01, ^∗∗∗^*P* < 0.001.

RTFs were isolated from the tracheal tissues and validated by immunofluorescence staining for α-SMA and vimentin. The expression of vimentin was positive and that of α-SMA was negative (Fig. S1). The mRNA expression of SOX9 in RTF was detected at different concentrations (0, 3, 5, and 10 ng/mL) and time points (0, 24, and 48 h) of TGF-β1 treatment, and it was found that 10 ng/mL TGF-β1 treated RTF for 48 h significantly increased the mRNA expression of SOX9 (*P* < 0.05; Fig. 1A, B). It was also noticed that TGF-β1 (10 ng/mL for 48 h) significantly increased the mRNA levels of ACTA2, Col1α1, and FN1 (*P* < 0.05; Fig. 1C). The protein expression levels of SOX9, collagen1, and α-SMA were also found to be up-regulated following TGF-β1 treatment by WB (Fig. 1D, E). In addition, immunofluorescence staining confirmed that SOX9 was localized in the nucleus of RTF cells, and the expression of SOX9 and α-SMA was enhanced after TGF-β1 treatment (Fig. 1F, G). Based on the above results, we can speculate that the high expression of SOX9 may promote fibroblast activation and ECM deposition.

### SOX9 overexpression promotes RTF activation and ECM deposition

**Figure 2:**
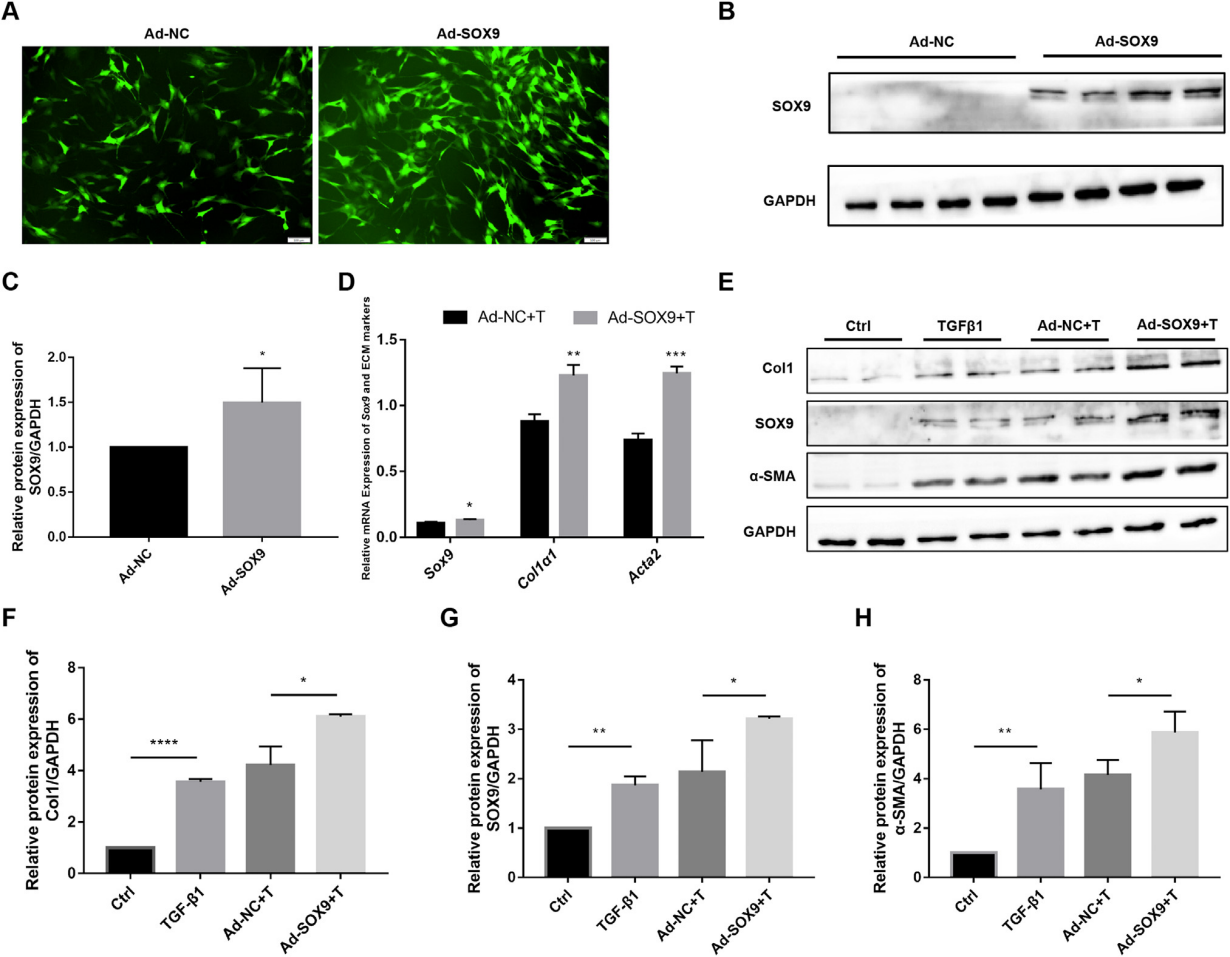
SOX9 overexpression promotes RTF activation and ECM deposition. RTFs were transfected with Ad-NC/SOX9. **(A)** Transfection efficiency was observed under a fluorescence microscope. Scale bars: 100 μm. **(B, C)** The transfection efficiency and quantitative analysis of SOX9 validated by Western blot. **(D)** The mRNA expression of *Sox9*, *Acta2*, and *Col1α1* in RTFs from the Ad-NC + T group and the Ad-SOX9 + T group determined by RT-qPCR. **(E)** The protein expression of SOX9, α-SMA, and collagen 1 in RTFs from the Ad-NC + T group and the Ad-SOX9 + T group determined by Western blot. **(F–H)** Quantification analysis of (E). ^∗^*P* < 0.05, ^∗∗^*P* < 0.01, ^∗∗∗^*P* < 0.001, ^∗∗∗∗^*P* < 0.000.

To determine whether ECM deposition can be modulated in a SOX9-dependent manner in RTF cells, and to further elucidate the effect of SOX9 on fibroblast activation and ECM deposition, we transiently transfected RTF cells with SOX9 overexpression adenovirus or NC adenovirus. The transfection efficiency was high, as verified by immunofluorescence staining and WB (Fig. 2A–C). It was found that the SOX9 overexpression significantly increased the mRNA and the protein expressions of SOX9, collagen 1, and α-SMA (*P* < 0.05; Fig. 2D–H). Therefore, SOX9 induction increased ECM deposition in RTF cells.

### SOX9 knockdown inhibits cell proliferation, migration, and fibroblast activation, reduces ECM deposition, and promotes apoptosis in TGF-β1-treated RTFs

**Figure 3:**
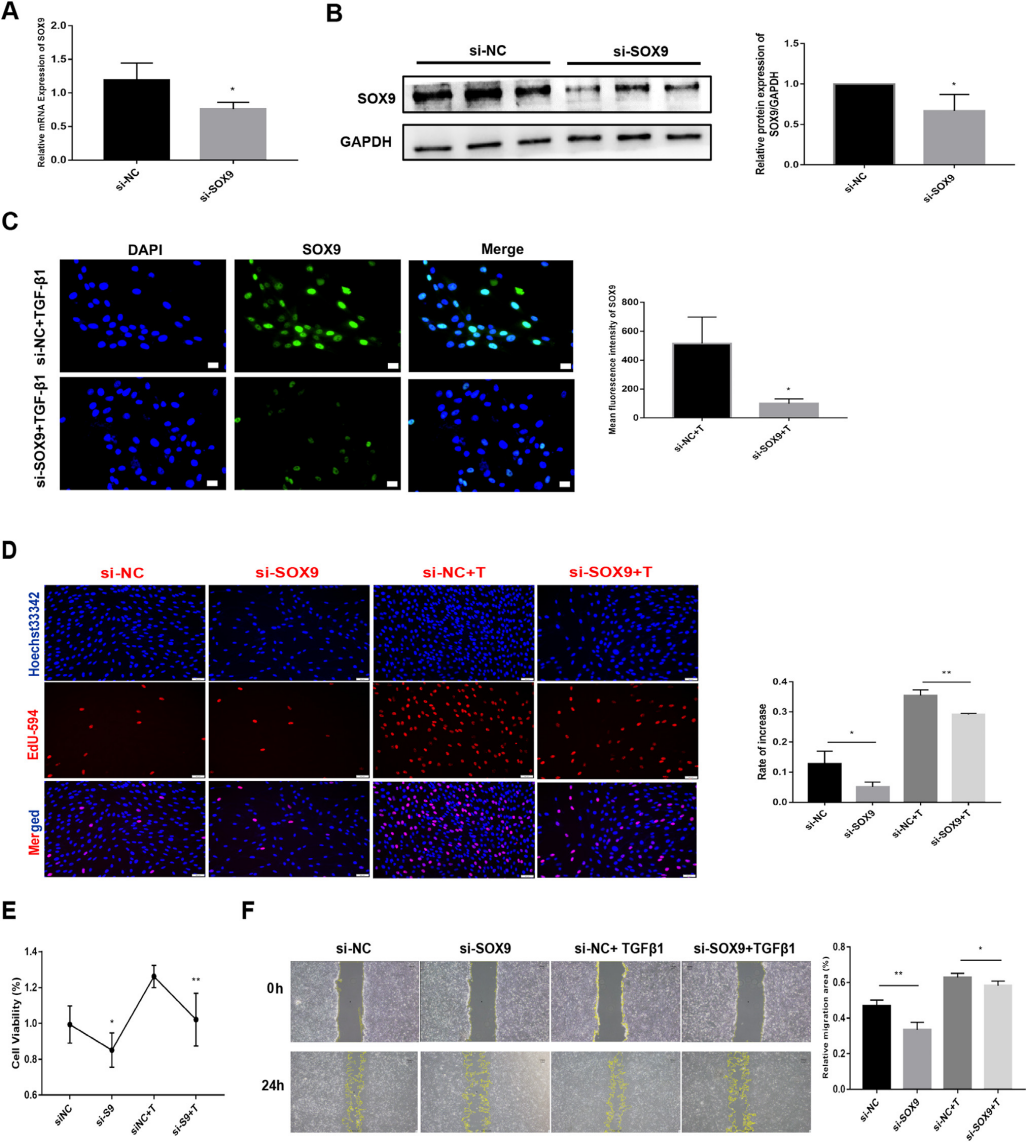
SOX9 knockdown inhibits cell proliferation and migration in TGF-β1-treated RTF. **(A)** The mRNA expression of *Sox9* in the si-NC and the si-SOX9 group was determined by RT-qPCR. **(B)** The protein expression and quantitative analysis of SOX9 in RTFs from the si-NC and the si-SOX9 group evaluated by Western blot. **(C)** Immunofluorescence staining of SOX9. Scale bars: 20 μm. **(D)** Cell proliferation and quantitative analysis in the sh-NC and the sh-SOX9 group with or without TGF-β1 treatment evaluated by EdU staining. Scale bars: 50 μm. **(E)** Cell viability in the si-NC and the si-SOX9 group with or without TGF-β1 treatment evaluated by CCK8. **(F)** Cell migration and quantitative analysis in the si-NC and the si-SOX9 group with or without TGF-β1 treatment evaluated by a wound-healing assay. Scale bars: 100 μm. ^∗^*P* < 0.05, ^∗∗^*P* < 0.01.

The knockdown efficiency of SOX9 in RTFs was verified by RT-qPCR and WB (Fig. 3A, B). After treatment with si-SOX9, the intensity of SOX9 fluorescence was attenuated even after TGF-β1 treatment (Fig. 3C). EdU staining and CCK8 results showed that upon TGF-β1 stimulation, cell proliferation was significantly diminished in the si-SOX9 group compared with that in the si-NC group with or without TGF-β1 treatment (*P* < 0.05; Fig. 3D, E). The migration ability of RTF cells was also diminished in the si-SOX9 group with or without TGF-β1 treatment (Fig. 3F).

**Figure 4:**
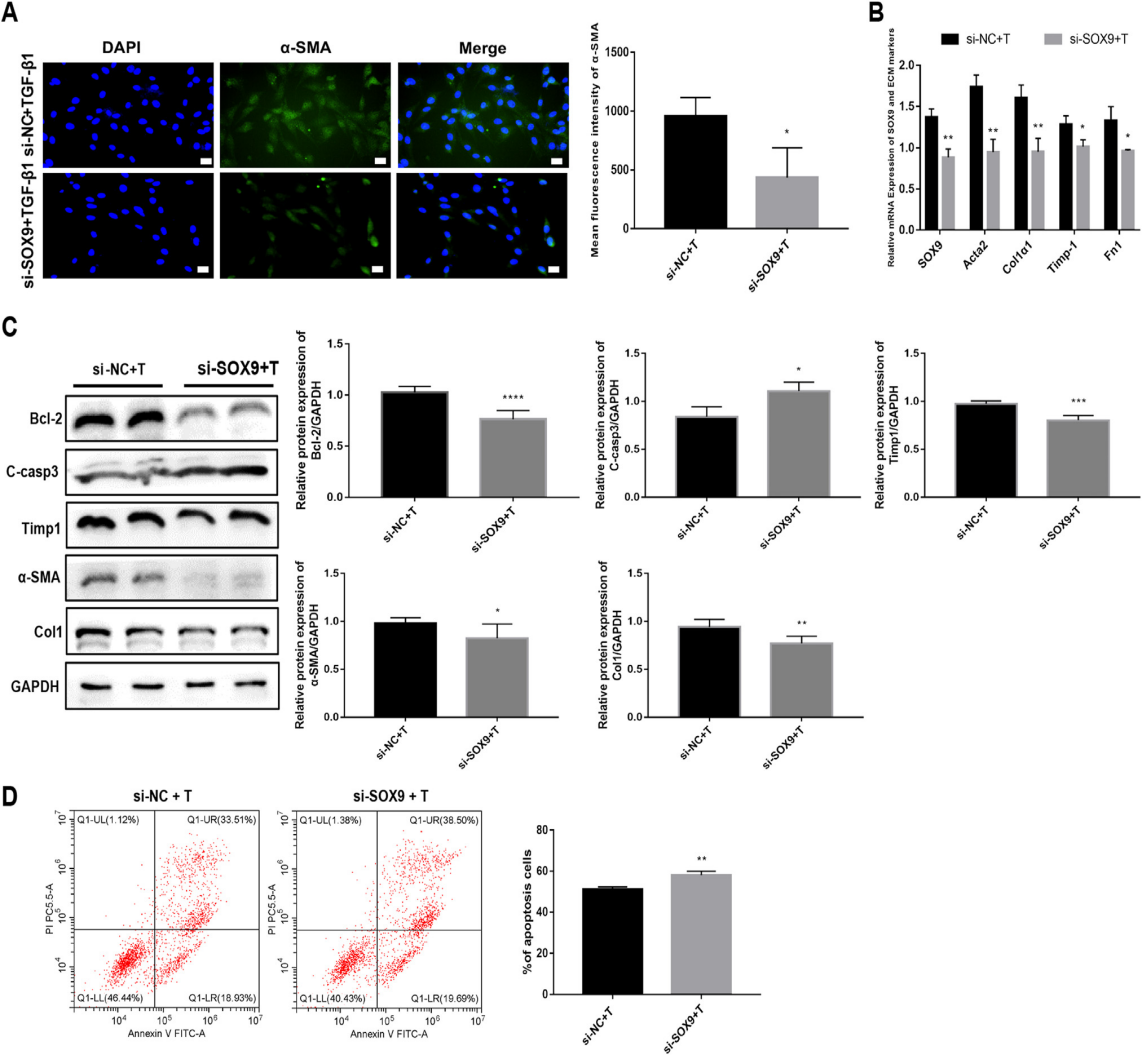
SOX9 knockdown inhibits fibroblasts' activation, reduces ECM deposition, and promotes apoptosis in TGF-β1-treated RTF. **(A)** Immunofluorescence staining and quantitative analysis of α-SMA. Scale bars: 20 μm. **(B)** The mRNA expression of *Sox9*, *Acta2*, *Col1α1*, *Timp1*, and *Fn1* in RTFs from the si-NC + T group and the si-SOX9 + T group determined by RT-qPCR. **(C)** The protein expression and quantitative analysis of Bcl-2, C-casp3, TIMP1, α-SMA, and Collagen1 in RTFs from the si-NC + T group and the si-SOX9 + T group evaluated by Western blot. **(D)** Apoptosis rates in the si-NC + T group and the si-SOX9 + T group assessed by flow cytometry. ^∗^*P* < 0.05, ^∗∗^*P* < 0.01, ^∗∗∗^*P* < 0.001, ^∗∗∗∗^*P* < 0.000.

The immunofluorescence results showed that SOX9 knockdown in TGF-β1-stimulated RTF cells dramatically inhibited fibroblast activation (Fig. 4A). The mRNA expression levels of SOX9, Acta2, Col1α1, Timp1, and Fn1 significantly decreased in the si-SOX9 group treated with TGF-β1 (Fig. 4B). The protein expression levels of fibrosis-related markers, such as Col1, TIMP1, and FN, were inhibited; the pro-apoptotic protein marker C-casp3 was increased; and the anti-apoptotic protein marker Bcl-2 was decreased in the si-SOX9 group treated with TGF-β1 (Fig. 4C). SOX9 knockdown increased the apoptosis rate of TGF-β1-treated RTF cells (Fig. 4D). In summary, these findings suggest that SOX9 knockdown can alleviate TGF-β1-induced cell proliferation, migration, and ECM deposition, and promote apoptosis in RTF cells.

### SOX9 transcriptionally regulates the expression of genes related to tracheal injury and repair

**Figure 5:**
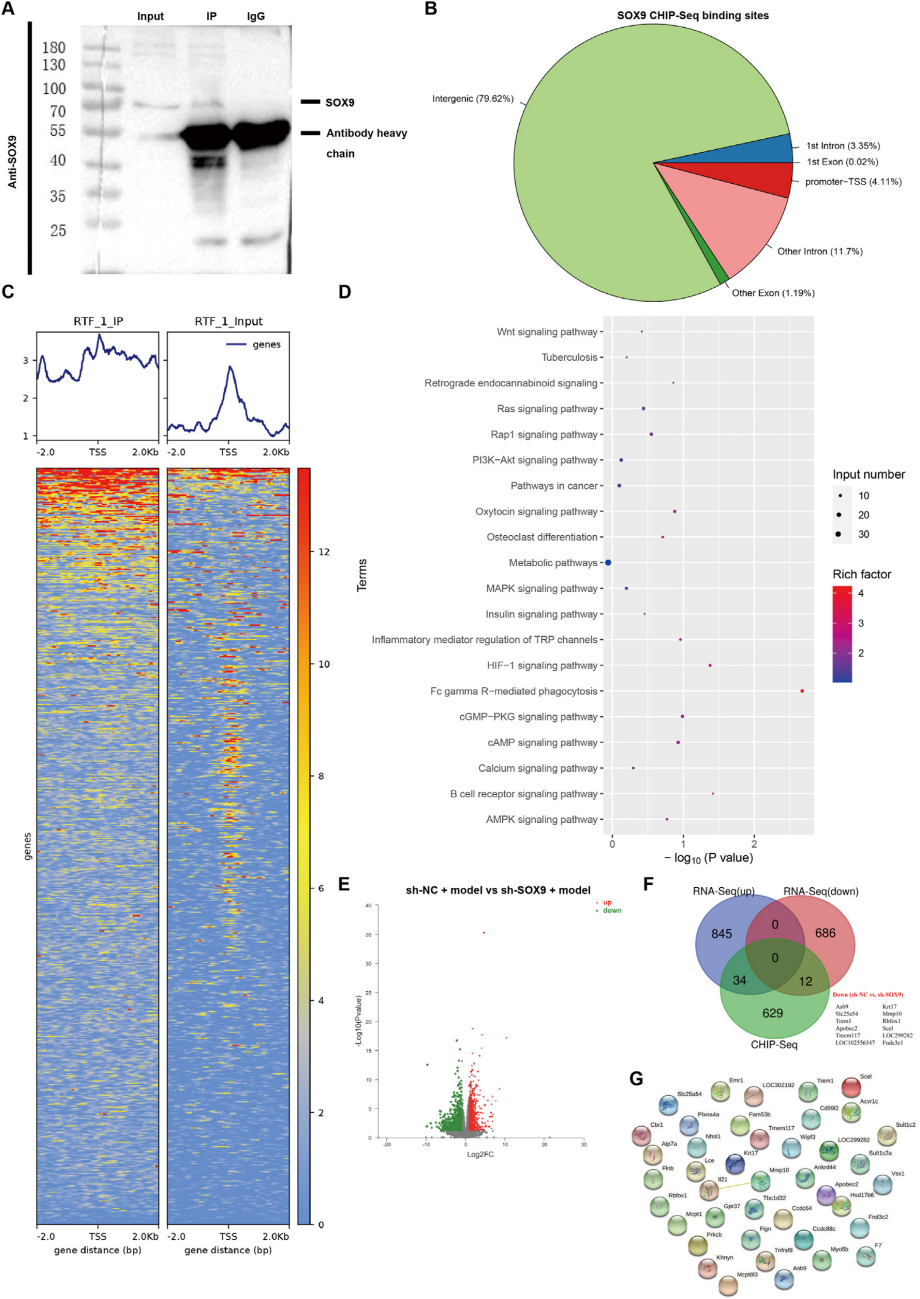
SOX9 transcriptionally regulates the expression of genes related to tracheal injury and repair. **(A)** Co-immunoprecipitation was carried out with anti-SOX9. **(B)** SOX9-binding distribution among intergenic, 1st intron, 1st Exon, promoter, and other exonic and intronic regions. **(C)** SOX9-enriched genes in RTF cells by ChIP-Seq analyses. **(D)** KEGG analysis of SOX9-bound genes. **(E)** The volcano plot displayed the differentially expressed genes in rat trachea tissues upon SOX9 knockdown by RNA sequencing (*n* = 5). **(F)** Venn diagram of a small subset (*n* = 12) of overlapping SOX9-responsive genes down-regulated in RNA sequencing and showed SOX9 peak in ChIP-sequencing. **(G)** The PPI network of the 12 genes hub genes was visualized with Cytoscape.

ChIP-seq was performed in RTF cells to identify genes directly targeted by SOX9. From the results of the IP-WB quality inspection, we can see that the Input and IP swimming lanes succeeded in detecting the target protein bands, and the IP experiment was successful (Fig. 5A). Peak calling by the analysis of the ChIP-seq algorithm revealed 675 transcription sites bound directly to SOX9, and the 675 genes contained SOX9-binding sites distributed in intergenic (79.82%), 1st intron, 1st exon, promoter (4.11%), and other exonic and intronic regions (Fig. 5B). SOX9-binding genes were distributed on both sides of the transcriptional initiation site (TSS) (Fig. 5C). KEGG analysis revealed that the Wnt/β-catenin signaling pathway is involved in the transcriptional regulation of SOX9 target genes (Fig. 5D). RNA extracted from the sh-SOX9 + and sh-NC + rat models was subjected to whole transcriptome sequencing (RNA-seq) analysis. A total of 1577 genes were differentially expressed between the two groups (Fig. 5E). We explored the molecular mechanisms underlying SOX9-induced biological effects in RTFs and rats. Interestingly, when we compared the 698 commonly down-regulated genes identified by RNA-seq and the genes with SOX9-bound transcription sites detected by ChIP-seq, we identified 12 overlapping genes, among which SOX9 showed the highest enrichment of bound SOX9 within its promoter and a significant change in RNA expression (Fig. 5F). Figure 5F shows that a small subset of genes (*n* = 12) was down-regulated in the shSOX9 + rat model and the ChIP-seq analysis of RTFs. The PPI network of the 12 hub genes was visualized using Cytoscape, and MMP10 was found to be a hub gene (Fig. 5G).

### SOX9 directly targets and transcriptionally activates MMP10 via direct binding to the SOX9 binding site

**Figure 6:**
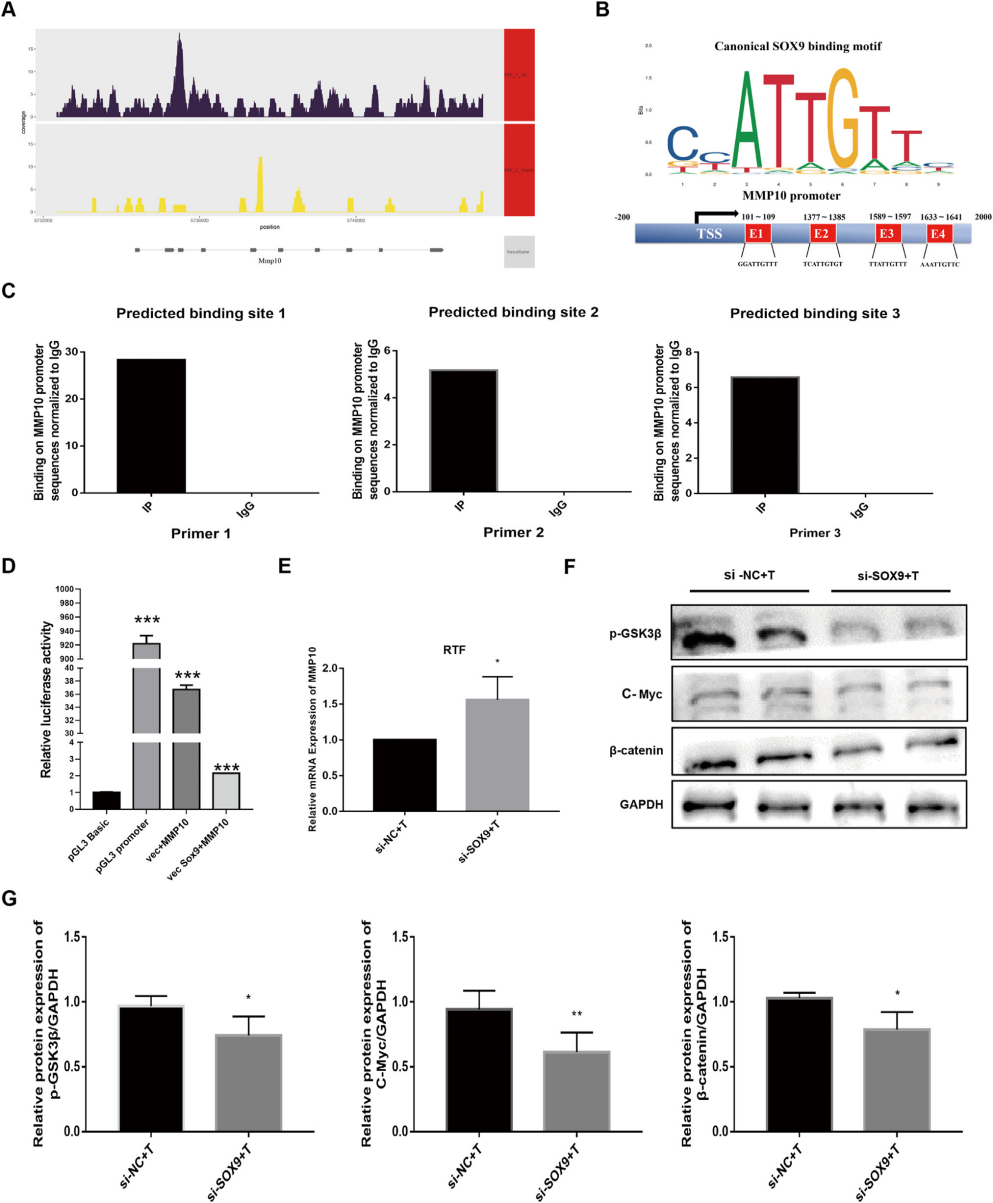
Profibrotic role of SOX9-MMP10-ECM biosynthesis axis in the repair of tracheal injury through the Wnt/β-catenin signaling pathway. **(A)** Example of peak associated with MMP10 in ChIP-seq and input DNA. **(B)** Schematic diagram of canonical SOX9-binding motif (JASPAR database) and three potential SOX9 responsive elements (E1, E2, and E3) in the MMP10 promoter region. TSS is the transcriptional start site of MMP10. **(C)** ChIP-qPCR validation of SOX9 binding site in the promoter of MMP10. **(D)** Relative Rluc/fluc activity of cells co-transfected with either Vector + MMP10 or Vector SOX9 + MMP10 and pGL3 basic or pGL3 promoter. **(E)** The mRNA expression of MMP10 in RTFs from the si-NC + T group and the si-SOX9 + T group detected by RT-qPCR. **(F, G)** The protein expression and quantitative analysis of p-GSK3β, c-Myc, and β-catenin in RTFs from the si-NC + T group and the si-SOX9 + T group evaluated by Western blot. ^∗^*P* < 0.05, ^∗∗^*P* < 0.01, ^∗∗∗^*P* < 0.001.

The upstream TSS of MMP10 was highly enriched for SOX9 binding (Fig. 6A). To test the results of ChIP-Seq, the possible binding sites of the transcription factors SOX9 and MMP10 were predicted using the JASPAR database. In the corresponding predictions, three highly rated binding sites were selected (Fig. 6B). Since MMP10 was reported to be a pivotal enzyme in ECM deposition that catalyzes the transformation of desmosterol to ECM, we explored whether SOX9 modulated ECM deposition through the regulation of MMP10. The SOX9 bound chromatin was analyzed by qPCR using primers for three regions within the MMP10 promoter that contained SOX9 binding sites. Our ChIP-qPCR results demonstrated that SOX9 could directly bind to the MMP10 promoter at the putative SOX9 binding site (Fig. 6C). The dual-luciferase reporter assays further showed that SOX9 caused a significant decrease in the luciferase activity of the MMP10 promoter–reporter construct, indicating that SOX9 inhibited MMP10 transcription (Fig. 6D). These results indicated that SOX9 directly targets and transcriptionally inhibits MMP10 by directly binding to the SOX9 binding site. To further evaluate the transcriptional regulation of SOX9 on MMP10, SOX9 knockdown by SOX9-specific si-RNA was performed in RTFs. As expected, the results of RT-qPCR showed that the enhanced expression of MMP10 was activated while knocking down SOX9 (Fig. 6E). To confirm the involvement of the Wnt/β-catenin pathway in the mechanism by which SOX9 promotes tracheal fibrosis, proteins related to the Wnt/β-catenin signaling pathway in RTF cells after SOX9 knockdown were measured. The results showed that SOX9 knockdown alleviated the expression of β-catenin, GSK3β phosphorylation (ser9), and c-Myc (Fig. 6F, G).

### SOX9 knockdown attenuates granulation tissue proliferation and fibrosis after tracheal injury in rats

**Figure 7:**
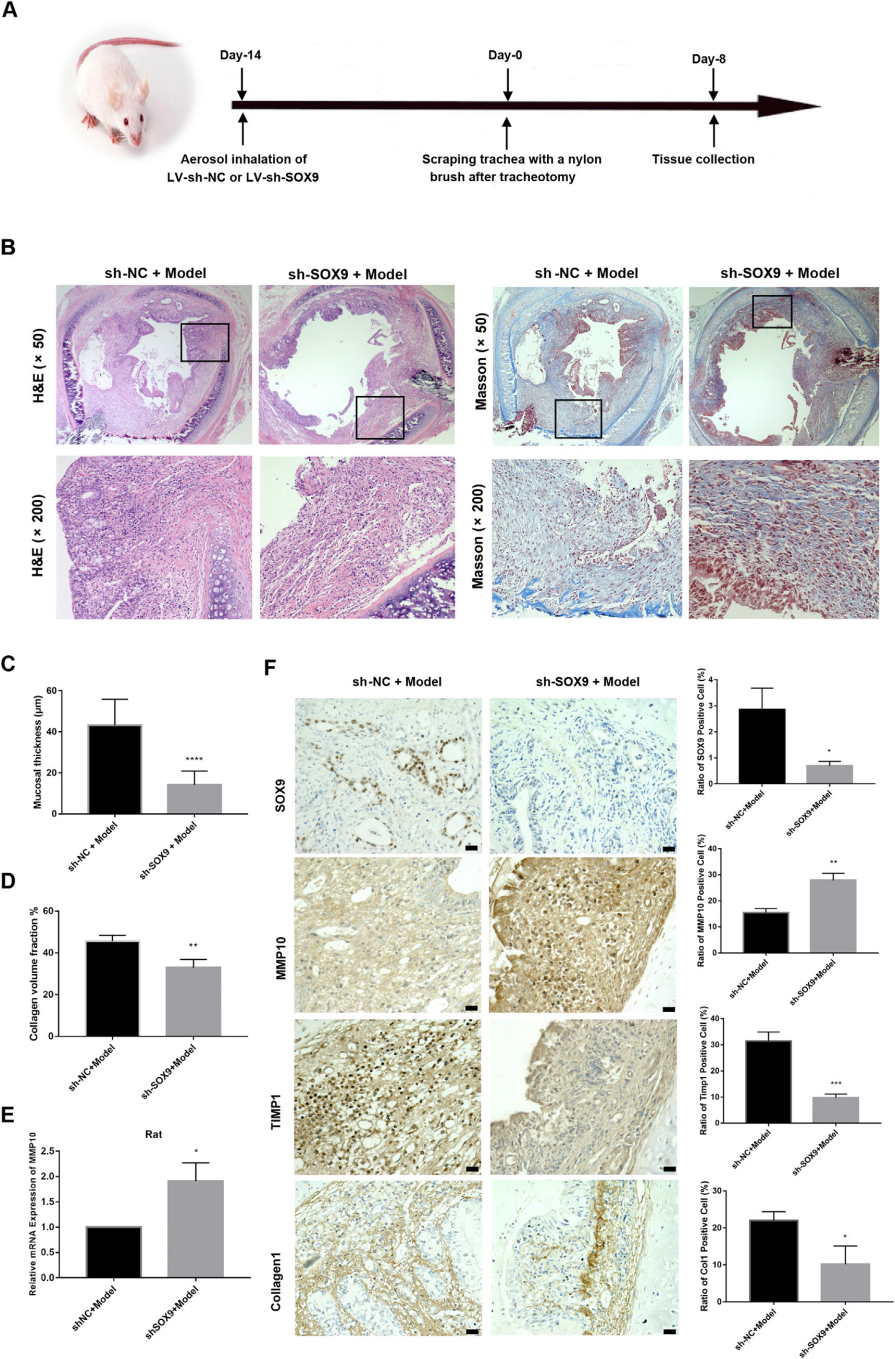
SOX9 knockdown attenuates granulation tissue proliferation and fibrosis after tracheal injury in rats through the SOX9-MMP10-ECM biosynthesis axis. **(A)** A general diagram of experimental design in rats. **(B)** H&E staining and Masson's trichrome staining of tracheal tissues (× 50, × 200). Scale bars: upper panels, 20 μm; lower panels, 50 μm. **(C)** Quantification of the tracheal mucosal thickness of (B). **(D)** Quantification of collagen volume fraction of (B). **(E)** The mRNA expression of MMP10 in rats from the sh-NC + model group and the sh-SOX9 + model group detected by RT-qPCR. **(F)** IHC staining and quantitative analysis of SOX9, MMP10, TIMP1, and collagen 1 in rats from the sh-NC + model group and the sh-SOX9 + model group. Scale bars: 20 μm.

**Figure 8:**
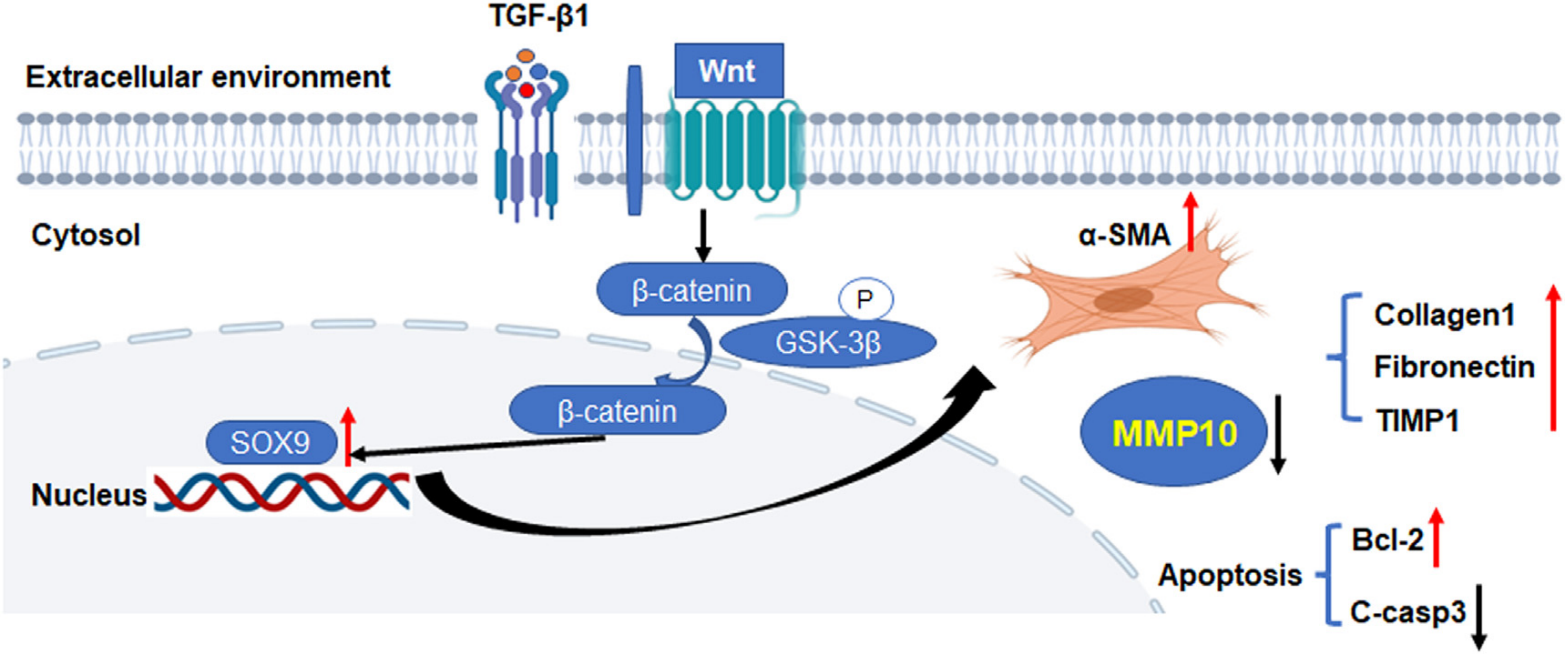
A mechanistic illustration of the role of SOX9-MMP10-ECM biosynthesis axis in fibroblast activation, cell apoptosis, and ECM deposition. The present study uncovers that TGF-β1 activates the Wnt/β-catenin signaling pathway and up-regulates SOX9 expression. High expression of SOX9 can activate fibroblast, resist cell apoptosis, and down-regulate MMP10 transcription to drive ECM deposition in RTF cells, consequently leading to fibrotic repair after tracheal injury. Pharmacological targeting of SOX9-MMP10-ECM may be a promising strategy to treat tracheal fibrosis.

To verify the role of SOX9 in the repair of tracheal injury, LV-shRNA-SOX9 and LV-shRNA-NC were transduced into rat trachea by nebulization for 2 weeks before modeling and on the eighth day after modeling, and the tracheal tissues were collected (Fig. 7A). The granulation tissue proliferation and fibrosis after tracheal injury in rats were assessed based on H&E staining and Masson's trichrome staining, and the tracheal mucosal thickness and collagen fiber deposition were decreased significantly when the expression of SOX9 was inhibited (Fig. 7B–D). The expression of MMP10 at the mRNA level was increased while knocking down SOX9 in the rat model of tracheal injury (Fig. 7E). Further, proteins related to the SOX9-MMP10-ECM biosynthesis axis were evaluated by IHC analysis, and we found that the enhanced expression of MMP10 was activated while knocking down SOX9, resulting in decreased expression of TIMP1 and decreased deposition of collagen 1 (Fig. 7F). Based on the above results, we revealed a novel mechanism that SOX9 inhibits MMP10 transcription by directly binding to its promoter region, which may, at least in part, reduce the degradation of excess ECM deposition after tracheal injury. Collectively, SOX9 acts as a positive regulator of ECM deposition by decreasing MMP10 expression. SOX9 knockdown alleviated TGF-β1-induced cell proliferation, fibroblast activation, and ECM deposition, and promoted apoptosis in RTF cells through the Wnt/β-catenin signaling pathway (Fig. 8).

## Discussion

The molecular mechanism underlying the association between SOX9 up-regulation and tracheal fibrosis remains unknown. Our novel findings demonstrated that SOX9 is aberrantly activated and up-regulated in RTF cells treated with TGF-β1. We demonstrated that SOX9 overexpression triggers fibroblast activation and promotes ECM deposition. In contrast, SOX9 knockdown suppressed TGF-β1-induced cell proliferation, migration, and ECM deposition and promoted the apoptosis of RTFs through the Wnt/β-catenin pathway. These results illustrate that TGF-β1 activates the Wnt/β-catenin signaling pathway and up-regulates the expression of SOX9, which down-regulates MMP10 transcription to drive ECM deposition, consequently leading to fibrotic repair after tracheal injury. Therefore, injury-induced tracheal fibrosis depends on SOX9-driven increases in profibrotic gene networks and fibroblast activation.

Multiple studies have shown that SOX9 plays a key role in cell proliferation, invasion, EMT, and ECM deposition in various organ fibrosis and related diseases.^17^, ^18^, ^19^, ^20^, ^21^, ^22^, ^23^ Liu et al^24^ analyzed and compared the RNA-seq data at different time points from acute kidney injury (AKI) to chronic kidney injury and found that the expression level of SOX9 remained high, even a few weeks after AKI, indicating that SOX9 is not only involved in the repair process but also in chronic fibrosis. Zhang et al^25^ found that SOX9 was up-regulated in renal tubular epithelial cells of rats with obstructive nephropathy. SOX9 overexpression promoted EMT, ECM deposition, and phosphorylated AKT up-regulation in renal tubular epithelial cells, while the PI3K inhibitor could reverse this phenomenon, indicating that SOX9 can promote renal fibrosis through the PI3K/AKT signaling pathway. Li et al^26^ showed that the knockdown or overexpression of SOX9 alleviated or exacerbated renal fibrosis, respectively. Fan et al^27^ found that inhibiting SOX9 significantly reduced the inflammatory response and liver cell apoptosis in mice after ischemia–reperfusion (IR). RNA sequencing of hepatic stellate cells (HSCs) revealed that 37% of SOX9-regulated genes were associated with ECM.^5^ Athwal et al^6^ found that SOX9 knockdown reduced hepatic inflammation levels, improved liver function, and inhibited liver fibrosis. In the heart, SOX9 is predominantly expressed in cardiomyocytes and cardiac fibroblasts after myocardial infarction in mice. Importantly, fibroblast-specific SOX9 deletion was sufficient to attenuate the migration, proliferation, and contractility of cardiac fibroblasts and to improve cardiac function.^28^

A previous study has identified increased expression of the BCL family of anti-apoptotic genes, including BCL-2, BCL-XL, and BCL2L2 in IPF fibroblasts.^29^ These genes contribute to the resistance against Fas-mediated apoptotic clearance in fibroblasts and myofibroblasts.^30^, ^31^, ^32^ Therefore, we analyzed the role of SOX9 in fibroblast survival. We found that SOX9 promoted fibroblast survival by inducing the expression of anti-apoptotic genes (BCL-2) and increasing the expression of pro-apoptotic genes (C-casp3). Thus, our results indicate that enhanced expression of SOX9 in tracheal fibrosis and TGFβ1-treated fibroblasts allowed the cells to resist Fas-mediated apoptosis. Consequently, the survival and accumulation of activated fibroblasts are likely to occur during tracheal fibrosis.

Likewise, we found that SOX9 knockdown attenuated migration and proliferation in RTF, further suggesting the importance of SOX9-positive fibroblasts as a part of the profibrotic response in multiple organs. Interestingly, SOX9 overexpression in RTF led to an increase in profibrotic factors such as α-SMA, collagen 1, and fibronectin, resulting in tissue remodeling. Our studies suggest that SOX9 regulates the expression of these profibrotic factors in myofibroblasts, either directly or indirectly, and likely forms a positive feed-forward loop, resulting in progressive fibrosis.

Guo et al^33^ found that SOX9 can promote survival, proliferation, invasion, and EMT, but these promotions are weakened after the knockout of the *MKLN1-AS* gene, and the overexpression and silence of SOX9 can increase or decrease the expression of *MKLN1-AS* in hepatocellular carcinoma cells, which results from the fact that SOX9 can transcriptionally regulate lncRNA-MKLN1-AS. In our study, SOX9 knockdown in RTF cells reduced migration and down-regulated several ECM-related genes. Using SOX9 silencing and overexpression strategies in fibroblasts, we demonstrated that SOX9 functions as a positive regulator of migration, proliferation, and ECM deposition. However, the SOX9-driven gene networks in fibroblasts that cause the pathological features observed in tracheal fibrosis are poorly defined. Based on ChIP-seq data, we found that the target genes regulated by SOX9 were enriched in injury and repair. In combination with our RNA-seq data, we identified MMP10, a matrix metalloproteinase in ECM biosynthesis that catalyzes ECM degradation, as a SOX9 target for transcription activation.

These results demonstrated that targeting SOX9 and its downstream targets can reduce the inflammatory response after injury, reduce ECM deposition, and inhibit or prevent tracheal fibrosis after injury. Overall, our findings indicate that SOX9 positively regulates fibroblast activation by inducing migration, proliferation, survival, and production of ECM in tracheal fibrosis. It is important to note that multiple growth factors can up-regulate SOX9 in fibroblasts and epithelial cells.^34^^,^^35^ Therefore, we cannot rule out the possibility that other mechanisms may be involved in SOX9-induced tracheal fibrosis. While we implicated SOX9 in fibroblast activation and tracheal fibrosis, further studies are necessary to determine the impact of SOX9 in other tracheal cells, including epithelial cells, macrophages, and other immune cells during the initiation, maintenance, and resolution of tracheal fibrosis.

Taken together, our study is the first to demonstrate the profibrotic activity of SOX9 in fibroblast activation, proliferation, ECM deposition, and cell apoptosis in RTF cells through activating Wnt/β-catenin signaling. The expression of SOX9 can be up-regulated by activating the Wnt/β-catenin signaling, which in turn inhibits MMP10 expression and promotes ECM deposition. Therefore, we identify a novel role of the SOX9–MMP10–ECM deposition axis in tracheal fibrosis. Pharmacological targeting of SOX9 with selective SOX9 inhibitors may be a promising strategy to treat tracheal fibrosis.

## Author contributions

The concept and design of the present study were provided by SLG and YSL. LG, AML, and CYH made substantial contributions to carry out the experiments. YSL, XHW, JYJ, YB, XXJ, MLX, XZ, TRT, and YX collected and processed clinical samples for RTF cell culture. JXL and RX helped to carry out the experiments. Experiment data were collected and analyzed by LX, MJY, JHM, and JL. LG produced the manuscript. Finally, SLG and YSL conducted data auditing and manuscript review. All authors read and approved the final manuscript.

## Conflict of interests

The authors declare no competing for financial interest.

## Funding

This study was supported by the National Natural Science Foundation of China (No. 82370098), 10.13039/501100013076National Major Science and Technology Projects of China (No. 2018ZX10302302003), Chongqing Talents projects-Famous Masters and Teachers (Shuliang Guo), Chongqing Young and Middle-aged Medical High-end Talent Studio – Lung Nodule Studio, and the Postgraduate Research and Innovation Project of Chongqing, China (No. CYB21173).
