# Supplementary material for: Profibrotic role of the SOX9–MMP10–ECM biosynthesis axis in the tracheal fibrosis after injury and repair
Source: Genes Dis. 2023 Jul 15;11(5):101040. doi: 10.1016/j.gendis.2023.06.012 (PMC11237849; doi:10.1016/j.gendis.2023.06.012)
Supplement: Multimedia component 1 [file mmc1.docx]

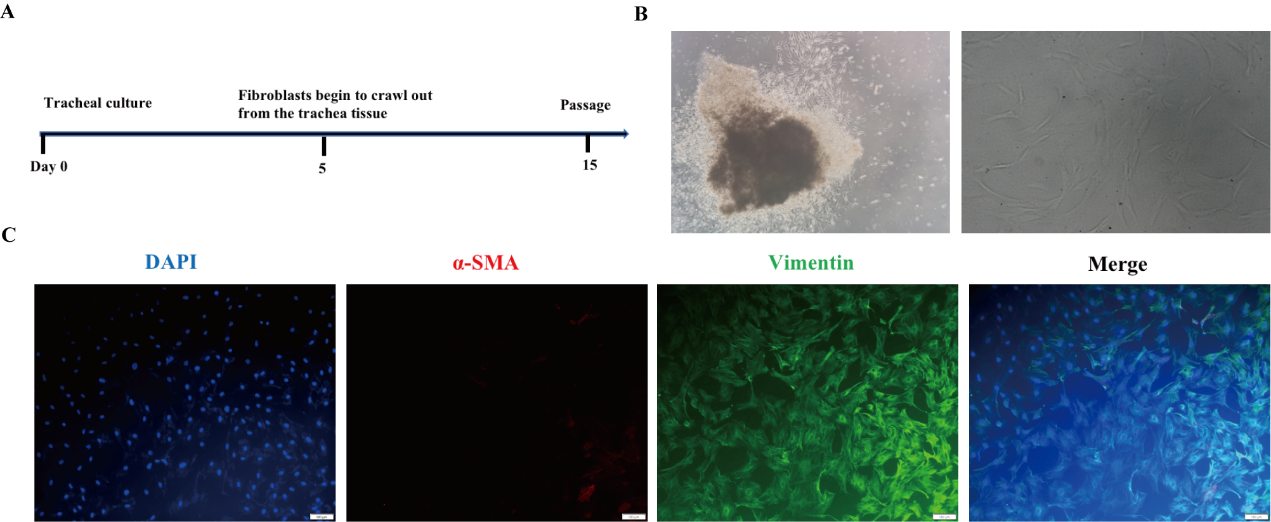


**Figure S1. Isolation and identification of primary RTF.** (A-B) Schematic representation of isolation, and culture of primary RTF. (C) Immunofluorescence co-staining of α-SMA and Vimentin confirmed RTF cells (×200).
