# Supplementary material for: Profibrotic role of the SOX9–MMP10–ECM biosynthesis axis in the tracheal fibrosis after injury and repair
Source: Genes Dis. 2023 Jul 15;11(5):101040. doi: 10.1016/j.gendis.2023.06.012 (PMC11237849; doi:10.1016/j.gendis.2023.06.012)
Supplement: Multimedia component 2 [file mmc2.docx]

**RNA isolation and RT-qPCR**

The RNA from RTF cells was isolated using an RNeasy kit (Takara Bio, Inc.). Subsequently, the fluorescence quantitative polymerase chain reaction (qPCR) analysis was performed using the CFX96 real‑time PCR system (Bio‑Rad Laboratories, Inc.) and the TB Green Premix Ex Taq II PCR kit (Takara Bio, Inc.) to amplify the target genes and internal reference genes. With beta-actin as the internal reference, the fold changes were calculated using relative quantification (2^-ΔΔCt^ method){Livak, 2001 #2724}[[1](#_ENREF_1)]. Primers for *beta-actin*, *Sox9*, *Acta2*, *Col1α1*, *Fn1*, *Timp1*, and *MMP10* were purchased from Takara Bio, Inc. The primer sequences are listed in *Table S1*. All experiments were repeated three times or more.

**Western blot**

The proteins extracted from cells were incubated with primary antibodies against GAPDH (cat.BM3874, 1:2500, BOSTER), SOX9 (cat.ab185966, 1:5000, Abcam), α‑SMA (cat.YM3245, 1:3000, Immunoway), Col1 (cat.AF7001, 1:1000, proteintech), Timp1 (cat.WL02342, 1:1000, Wanleibio), Bcl-2 (cat.WL01556, 1:500, Wanleibio), Cleaved Caspase-3 (cat.WL02117, 1:500, Wanleibio), C-Myc(cat.WL01781, 1:500, Wanleibio), β-catenin (cat.ab32572, 1:5000, Abcam), GSK-3β (cat.ab32391, 1:5000, Abcam), and p-GSK-3β (cat.AF2016, 1:1000, Affinity).

**Immunofluorescence (IF) staining**

RTF cells were fixed with 4% paraformaldehyde for 30 min, treated with 0.1% Triton-X for 10 min, and blocked with normal goat serum for 30min. The primary antibody used included SOX9 (cat.ab185966, 1:1000, Abcam), α-SMA (cat.ab7817, 1:100, Abcam). The next day, samples were incubated with the corresponding Alexa Fluor® 488-conjugated or Cy3-conjugated secondary antibodies, and DAPI. Images were then taken using an Olympus fluorescence microscope.

**Flow cytometry**

We pretreated cells with trypsin and fixed them at 4°C in 75% ethanol overnight, then we stained cells

with propidium iodide (PI) at 25 °C for 30min for cell cycle analysis. Apoptosis rate was detected by Annexin V/PI double staining (Sungene, China) under the instructions provided by the manufacturer. RTFs in each group were collected and resuspended in PBS, labeled with Annexin V-FITC and PI, and then incubated for 15 min in the dark. Subsequent analysis was performed on a FACS flow cytometer by using Cell Quest ver. 4.02 (Becton Dickinson, China).

**Cell counting kit‑8 assay**

Cell viability was evaluated with a CCK-8 kit (MedChem Express). 0.5×10^4^ RTF cells were seeded in a 96-well plate and treated as previously described. 10μl CCK-8 solution was added to each well 2h before the endpoint of incubation. Absorbance at 450 nm was measured via a microplate reader (Olympus, Tokyo, Japan).

**Wound-healing assay**

Cells were seeded at 8×10^4^ cells/well into 6-well plates and incubated until they reached 100% confluence. Then, the tip of a pipette was used to damage the cell monolayer. PBS was added to wash the wells, and DMEM was added. Wound healing was observed under an IX51 microscope (Olympus) every 12 h, and images were collected. The percentage of wound healing was calculated as an index to determine the cell migration ability.

**5-ethynyl-2'-deoxyuridine (EdU) staining**

EdU staining was performed with the BeyoClick EdU Cell Proliferation Kit with Alexa Fluor 594 (Beyotime, Shanghai, China). RTF cells were incubated with 10μM EdU for 2h, fixed with 4% paraformaldehyde for 15min, washed with PBS for 5min, and then incubated with permeate for 15min. After being washed for 5min, cells were incubated with the Click Additive Solution and Hoechst in the dark. Edu staining positive cells were then captured under a fluorescence microscope (Olympus, Tokyo, Japan).

**Reference**

1. Livak KJ, Schmittgen TD (2001) Analysis of relative gene expression data using real-time quantitative PCR and the 2(-Delta Delta C(T)) Method. Methods 25: 402-408. DOI 10.1006/meth.2001.1262
