# Supplementary material for: Profibrotic role of the SOX9–MMP10–ECM biosynthesis axis in the tracheal fibrosis after injury and repair
Source: Genes Dis. 2023 Jul 15;11(5):101040. doi: 10.1016/j.gendis.2023.06.012 (PMC11237849; doi:10.1016/j.gendis.2023.06.012)
Supplement: Multimedia component 3 [file mmc3.docx]

**Table S1. Primers used in the present study**

| Genes | Sequences |
| --- | --- |
| rat-*β-actin* | F: 5' GGAGATTACTGCCCTGGCTCCTAGC 3' |
|  | R:5' GGCCGGACTCATCGTACTCCTGCTT 3' |
| rat-*Sox9* | F: 5' CACCACTCCCAAAACAGACG 3' |
|  | R: 5' GGCAGGTATTGGTCAAACTCA 3' |
| rat-*Acta2* | F: 5' GCATCCGACCTTGCTAACG 3' |
|  | R: 5' CTCCAGAGTCCAGCACAATACC 3' |
| rat-*Col1α1* | F: 5' GCCTCAGAAGAACTGGTACATCA 3' |
|  | R: 5' GAACCTTCGCTTCCATACTCG 3' |
| rat-*Fn1* | F: 5' CCAAGTATGAAGTCAGCGTCTATG 3' |
|  | R: 5' TGATGGTAGTTTCTGTAGCGTCG 3' |
| rat-*Timp1* | F: 5' CCTCTGGCA TCCTCTTGTTG 3' |
|  | R: 5' CATCTTGA TCTCA TAACGCTGG 3' |
| rat-*MMP10* | F: 5' TGCTGCTGTGCTTTCCGAT 3' |
|  | R: 5' AGCAAGATCCATGGTTGAGTGG 3' |

**Abbreviations:** F, forward; R, reverse; rat, Rattus norvegicus
